# Functional Characterisation of Rosemary‐Enhanced Alginate Films for Strawberry Preservation

**DOI:** 10.1155/ijfo/5597893

**Published:** 2026-05-21

**Authors:** Laura Mitrea, Bernadette-Emöke Teleky, Silvia-Amalia Nemeş, Mihaela-Ştefana Păşcuţă, Adrian-Gheorghe Martău, Lavinia-Florina Călinoiu, Alina-Lăcrămioara Nistor, Carmen-Rodica Pop, Ancuţa-Mihaela Rotar, Francisc-Vasile Dulf, Bianca-Eugenia Ştefănescu, Magdalini Krokida, Dan-Cristian Vodnar

**Affiliations:** ^1^ Faculty of Food Science and Technology, University of Agricultural Sciences and Veterinary Medicine Cluj-Napoca, Cluj-Napoca, Romania, usamvcluj.ro; ^2^ Institute of Life Sciences, University of Agricultural Sciences and Veterinary Medicine Cluj-Napoca, Cluj-Napoca, Romania, usamvcluj.ro; ^3^ CENCIRA Agrofood Research and Innovation Centre, Cluj-Napoca, Romania; ^4^ Faculty of Agriculture, University of Agricultural Sciences and Veterinary Medicine Cluj-Napoca, Cluj-Napoca, Romania, usamvcluj.ro; ^5^ Department of Pharmaceutical Botany, Iuliu Hatieganu University of Medicine and Pharmacy, Cluj-Napoca, Romania, umfcluj.ro; ^6^ National Technical University of Athens, Athens, Greece, ntua.gr

**Keywords:** alginate edible coating, antimicrobial films, aqueous rosemary extract, distillation by-products, strawberry preservation, sustainable packaging

## Abstract

This study developed alginate‐based edible films enriched with rosemary extract (RE), obtained from distillation by‐products and evaluated their physicochemical, functional and preservation performance in strawberries. RE (9% *v*/*v*) was incorporated into 2% (*w*/*v*) sodium alginate film‐forming solutions, which were applied both as coatings and cast into standalone films. The incorporation of RE increased film thickness (from ~0.056 to ~0.068 mm), density (from ~1.05 to ~1.12 g/cm^3^) and solution viscosity, while reducing moisture content (from ~12% to ~8.5%). RE also enhanced UV–Vis light protection and slightly increased water vapour permeability (from ~2.75 to ~3.0 × 10^−11^ g · s^−1^ · m^−1^ · Pa^−1^). When applied to fresh strawberries stored at 5°C, RE‐enriched coatings significantly inhibited microbial growth. Total bacterial counts remained below 4.9 log10 CFU/g after 14 days compared with higher levels in uncoated controls, whereas total yeast and mould counts decreased from ~3.7 to ~3.1 log10 CFU/g. These results demonstrate that RE‐infused alginate films effectively slow microbial proliferation and help maintain fruit quality during refrigerated storage. The findings point out the efficacy of rosemary distillation by‐products as sustainable, natural additives for biodegradable edible coatings in fresh produce preservation.

**Practical Application**

• The conclusions of the current research prove the capacity of a seaweed‐derived biopolymer, namely alginate, incorporated into active packaging enriched with aqueous rosemary extract (RE) as a natural and maintainable combination for conserving very easily spoiling fruits like strawberries.
• By decreasing the growth of microbial entities and helping maintain fruit quality during refrigerated storage, these coatings may support the fresh produce industry in extending shelf life, reducing postharvest losses and decreasing reliance on synthetic preservatives.
• Additionally, drawing on rosemary distillation remainder as a base of bioactive compounds provides a valorisation pathway for agro‐industrial waste, aligning with circular economy and sustainable packaging strategies.
• The food‐grade, low‐cost nature of the materials and their compatibility with existing coating technologies further support their potential for industrial implementation.

## 1. Background

One of the most representative members of the Lamiaceae family, expressly rosemary (*Rosmarinus officinalis* L.), is largely recognised for its natural antioxidant and antimicrobial characteristics and has long been used in food preservation to inhibit oxidation and microorganisms contamination [1]. In recent years, rosemary–derived compounds have been increasingly infused within comestible pellicles and protective coverings in order to enhance the foodstuffs lifespan [2]. Despite these advances, most existing studies focus primarily on essential oils extracted from fresh plant material, whereas comparatively limited consideration has been given to the valorisation of rosemary distillation side‐products as alternative bases of bioactive principles. These residues remain rich in phenolic constituents, such as rosmarinic acid, which retain significant preservative potential [1]. Addressing this gap, this current study explores the recovery of bioactive compounds from rosemary distillation waste through aqueous extraction and their incorporation into biodegradable alginate‐based coatings. In this way, the study not only participates in ecological packaging elaboration but similarly promotes the circular utilisation of agro‐industrial by‐products, evaluating the effectiveness of the resulting films in preserving highly perishable fresh fruits such as strawberries.

The well‐known alginate, derived from brown algal cell walls, possesses fine film‐forming properties that are beneficial in creating edible coatings that prevent moisture loss and minimise gas exchange, which are essential factors in keeping the quality features of foodstuff while stowaging [3–5]. Furthermore, this biopolymer is both green‐labelled and nonhazardous compared with synthetic coatings, positioning it as a suitable candidate designated for ecological food wrapping alternatives. Responding to consumer preferences for vegan and eco‐friendly products, these seaweed‐derived edible coatings can incorporate various plant extracts to enhance their health benefits and functional attributes [6, 7]. In particular, essential oils or aqueous extracts resulting from aromatic plants like oregano and rosemary can offer increased antimicrobial properties and improve the antioxidant activity. As an example, their addition enhances the preservative effect of alginate coatings by inhibiting microbial spoilage [8, 9]. This synergy, which arises from the strong antioxidant properties of these natural compounds, working alongside alginate′s barrier capabilities, can extend the storage time of delicate foodstuffs, including plant and animal‐derived food products, such as agricultural stuff and meats [10, 11]. Thus, further research focusing on optimising the compositions of such coatings could yield significant benefits for food preservation industries.

Building on the properties discussed above, the primary target of the present research was to create sustainable alternatives to plastic packaging that use biodegradable materials to lengthen the preservation period of perishable goods. Explicitly, the current research is aimed at assessing the bioactive properties of RE polyphenols integrated within an alginate‐derived solution for covering and protecting highly perishable fruits. Additionally, the alginate‐based solution was analysed for opacity and viscosity. Several physical aspects of the firm films, such as heaviness, breadth, compactness and width, were assessed. This research also examined wetness, water dissolution and water vapour permeability and explored differences amongst films supplemented with glycerol as a plasticiser and those deprived of it for the extract variant used. Moreover, a novel aspect of this research lies in the assessment of microbiological quality over an extended storage period, providing quantitative insights into the antimicrobial efficacy of RE within alginate‐based coatings. Unlike previous studies that primarily focus on physicochemical properties, this study examines the progression of microbial growth on fresh fruits over 2 weeks, providing valuable data for real‐world applications in food preservation. Collectively, these efforts strengthen the case for RE‐infused bio‐coatings serving as a feasible alternative to synthetic preservatives in food packaging. Although alginate‐based coatings and RE have been studied individually, the specific integration approach in this study offers a novel functional synergy. Our formulation targets to optimise the retention and controlled release of rosemary bioactives within the alginate matrix, with particular emphasis on the integration of aqueous bioactives derived from distillation residual biomass and their integrated functional characterisation.

## 2. Resources and Procedures

### 2.1. Vegetal Matrix and Reagents

An essential oil producer in Volos, Greece, provided rosemary leaves. The biomass by‐product recovered from the plant fragments used in the extractions from the process of rosemary distillation. Sodium alginate (provided by BioChemica‐AppliChem, Barcelona, Spain) of analytical and food quality was employed as the seaweed‐derived biopolymeric matrix. The material was specified by the manufacturer as a medium‐viscosity grade suitable for film‐forming applications. Sourced from Merck (Darmstadt, Germany), glycerol was added as a plasticiser. All reagents serving to chemical analyses, including liquid chromatography (HPLC) antioxidant properties, dissolution in water and for the penetrability of water vapour (WVP) assessments, were of high‐purity quality and acquired from Merck (Darmstadt, Germany).

### 2.2. Water‐Based Extraction of Polyphenols From Rosemary

The biomass side‐products were dried, milled into a fine powder, sieved through a 500 *μ*m mesh to ensure homogenous particle dimension, and stored in a dry place until use. Further, 3 g of grounded specimen was blended into 30 mL purified H_2_O (1:10, *w*/*v*), then vortexed for 60 s. The blend was ultrasonic treated for about an hour at 23°C and subjected to centrifugation at 10.000 rpm (for maximum 10 min). The upper phase, consisting of the separated polyphenolic compounds, was clarified via a 0.45 *μ*m pore sized paper filter, then stored until further use. Aqueous extraction was chosen because it provides a food‐grade, safe and environmentally friendly method compatible with edible coatings, avoiding organic solvents while still recovering phenolic compounds with demonstrated antioxidant and antimicrobial activity. Although this approach primarily extracts hydrophilic polyphenols and may leave some liposoluble compounds in the residue, previous studies have shown that these water‐soluble compounds are sufficient to confer functional benefits in alginate‐based coatings [12]. Based on the previous studies, the aqueous phenolic extract that was further added to the alginate matrix was established at 9% of the total volume. Sequential extraction with alcohol could recover additional liposoluble bioactives for exploration in future studies.

### 2.3. The Chromatographic Analysis (HPLC‐MS) of Polyphenols in Rosemary Samples

The analysis was supported by means of a liquid chromatograph (model HP‐1200) fitted out with a degasser, quaternary pumping system, auto‐sampling unit, UV–Vis diode array detector and MS‐6110 single‐quadrupole mass spectrometer with atmospheric pressure ionisation–electrospray interface (provided by Agilent Technologies, United States). Polyphenols were analysed in positive ionisation mode, using fragmentor voltages in the range of 50–100 V. The chromatographic column was a Kinetex XB‐C18 (150 × 4.6 mm, 5 *μ*m particle size, Phenomenex, United States) [13, 14].

The moveable phase consisted of water acidified with 0.1% formic acid (A) and acetonitrile acidified with 0.1% formic acid (B). The sequential steps undeviating gradient was settled: begin at 5% phase B for about 120 s; ramp from 5% to 90% B over 20 min; maintain at 90% B up to 4 min, then ramp back to 5% B over 6 min. The investigation period was half an hour, the output frequency was 0.5 mL/min and the heater value was maintained at 25^°^C ± 0.5^°^C.

Mass spectrometric exposure of positively charged ions was accomplished by means of the scan mode. The investigational settings were established as follows: gas temperature 350°C, nitrogen flow rate 7 L/min, nebuliser pressure 35 psi, capillary voltage 3000 V, fragment 100 V and *m*/*z* 120–1500. Chromatographic outcomes were noted down at wavelengths *λ* = 280 nm and *λ* = 340 nm, and data acquirement was performed with the Agilent ChemStation software, Version B.02.01 SR2.

### 2.4. Film‐Forming Process

As control groups, film mixtures were prepared through formulating initial base mixtures, such as alginate (noted as A), and alginate supplemented with glycerol (noted as *A* + Gly). These consisted of 2% alginate and 1% glycerol, solubilised in H_2_O at elevated temperature (80°C) while stirring continuously for 120 min. RE was added to these foundational solutions, creating two additional formulations: *A* + RE and *A* + Gly + RE. The extracts were stirred constantly for a quarter of an hour at lower heat values (below 40°C). The aqueous RE was incorporated at 9% (*v*/*v*) of the total formulation, corresponding to approximately 1.35 mL extract per 15 mL of film‐casting mixture. Successively, 15 mL of each film mixture was poured in 90 mm Petri plates and let to harden at 21°C–23°C for 48–72 h. The solidified films have been removed and kept between layers of paper under ambient conditions for future analysis.

### 2.5. Film Examination

Different analyses on both liquid and solid forms of the film were performed as follows.

#### 2.5.1. Film Mixtures

UV–VIS absorbance at 600 nm was measured by means of a NanoDrop ND‐1000 UV–Vis spectrophotometer (Thermo Fisher Scientific, Massachusetts, United States). Each measurement was done in duplicate. Transparency (opacity) at 600 nm (T600) was established using the subsequent formula:

(1) T600600 nm/mm=A/b

*A*600 represents absorbance value at 600 nm and *b* represents coating width (mm).

The RE antioxidant capacity was evaluated by means of the DPPH (1,1‐diphenyl‐2‐picrylhydrazyl) free radical scavenging method described by Brand‐Williams et al. [15]. Duplicates of 35 *μ*L samples of the solutions were blended with 250 *μ*L of DPPH blend. The mixtures were maintained out of light at laboratory temperature (21°C–23°C) for 30 min, then spectral extinction was measured at 515 nm by means of a multimode microplate BioTek reader (Winooski, Vermont, United States). Outcomes were stated as micromole of equivalents of Trolox (*μ*mol TE) per 100 g of the sample [16].

Viscosity measurements of the seaweed‐derived specimens were performed by means of an Anton Paar MCR 72 rheometer (Anton Paar, Graz, Austria) fitted out with a P‐PTD 200/air Peltier plate‐plate system. Specimens were flanked by the two plates: the superior plate had a smooth parallel‐plate geometry with a 50 mm diameter, whereas the inferior plate included a heat‐control system fixed to four temperatures (10°C, 20°C, 30°C and 40°C) with a 0.1 cm gap between the plates. The surplus was taken off before measurements, and the specimens were permitted to rest for a few minutes to achieve heat value steadiness. Assessments were conducted under a proportionally ramped shear degree from 5 to 300 s^−1^. Viscosity measurements at multiple temperatures were performed to assess the flow behaviour and temperature sensitivity of the alginate‐based solutions, providing insight into coating uniformity, film formation and handling properties during processing.

#### 2.5.2. Compact Films

Physical assessments were performed: *Covering layer width* (*μ*m) was measured with a digital calliper (Lumytools LT15240, Suceava, Romania) in millimetres at five random points for individual film specimens, with each assessment duplicated. The *diameter* (mm) of each sample was also measured by means of the same calliper at three arbitrary points, and duplicate assessments were averaged. The *mass* (g) of the hard coatings was determined by means of an ALJ 220‐5DNM laboratory balance (accuracy of 0.0001 g), provided by Kern (Kern & Sohn Gmbh, Frankfurt, Germany). The mean value of two assessments was reported. The mass of every specimen was split by its area and width using the following calculation formula:

(2) Densityg/cm3=w/a·t

*w* represents mass of specimen (g), *a* signifies area of specimen (cm^2^) and *t* indicates width of specimen (cm). The final width was established as the mean of double assessments.

The moistness amount of the hard films was established via recording their mass previous and subsequent drying into a kiln at 110°C up to a steady weight was achieved. Each sample was analysed in duplicate. The equilibrium moistness amount (%) was established by means of the next calculation:

(3) Moisture content %=m0−m/m0100∗

*m*_0_ represents starting film mass (g) and *m* represents ending film mass (g).

The test for dissolution in water (WST) was achieved following the previous procedure proposed by Teleky et al. [12], with slight modifications. Specimens having a dimension of 4 cm^2^ were cut from every film and dehydrated inside of a desiccator containing calcium chloride for a few days (5 days). Mutually, film sections (*B*_0_) and filter sheet (*W*_0_: Whatman no. 1, size: 14.5 cm) were pondered (0.0001 g accuracy). At that point, the specimens were immersed in cups filled with 50 mL of deionized water and stirred for half an hour at 300 rotations, under laboratory conditions (23°C). Subsequent film blends were poured through Whatman sheets, then dehydrated 1 day at medium‐high temperature (50°C). The Whatman sheets have been chilled inside the desiccator and repondered (*W*_1_). Two assessments have been performed, and the proportion of whole solvable substance, stated as solubility (%), was established by means of the following calculation:

(4) WST%=B0−W10−W/B0100×

where *B*_0_ is the weight of film pieces (in grams) and *W*_1_ is the weight of Whatman filter papers before drying (in grams).

The transmission rate of water vapour penetrability (WVTR) (10^−11^·g·s^−1^·m^−1^·Pa^−1^) was performed by succeeding the same procedure described in our prior publication [17].

Additionally to WVTR, moisture content and water solubility were evaluated as complementary indicators of the films′ overall water barrier performance.

### 2.6. Film Solution Antimicrobial Efficacy Test by Application on Fresh Fruits

#### 2.6.1. Fresh Fruits Preparation and Storage Conditions

Strawberries were selected as perishable food items to test the antimicrobial efficacy of alginate‐based solutions enriched with RE. Fresh strawberries have been bought from a retail market (Cluj‐Napoca, Romania). Strawberries have been put into a temperature‐monitored atmosphere and maintained at 4°C for investigational purposes. The fruits were chosen based on even dimension, heaviness, colour and lack of external or pathological spoil, and positioned in sterile Petri dishes. The berries were thoroughly sprayed with the alginate‐based solution for a few minutes to confirm a constant covering. The berries were air‐desiccated at room temperature on a clean table for at least 1 h. Covered and uncovered fruits were set aside in uncovered sterile Petri dishes at 5°C (±0.5°C), 75% relative moisture for 14 days. All samples, as well as uncovered ones (blank samples), were evaluated as fresh and right after the covering (Day 0), at Day 7 and 14 days of storage. The tests were performed in duplicate.

#### 2.6.2. Microbiological Assessment of the Fresh and Coated Berries

##### 2.6.2.1. Total bacteria count (TBC)

The total amount of living bacteria (TBC) has been processed by means of the pour‐plate technique and plate count agar (PCA) as media, after SR EN ISO 4833‐1:2014/A1:2022 [18]. PCA (consisting of enzymatic digestion of casein 5 g/L; yeast extract 2.5 g/L; glucose anhydrous 1 g/L, agar 15 g/L) with an ending pH value of 7.0 ± 0.2 at 25°C has been utilised as a universal growing environment for identifying specific microbial entities, which could be found in fresh fruits (e.g., *Escherichia* spp., *Pseudomonas* spp., *Listeria* spp., *Salmonella* spp., *Staphylococcus* spp., *Streptococcus* spp. and *Yersinia* spp.). Subsequently, cultivated dishes have been maintained for about 2–3 days at 30°C. The outcomes have been reported as the logarithm of colony‐forming units per gram of the specimen (log_10_ CFU/g).

##### 2.6.2.2. Total Yeasts and Moulds Count (TYMC)

Spread plate methods and Dichloran Rose‐Bengal Chloramphenicol agar (DRBC) as media were employed to detect several fungi‐yeasts and moulds of significance in fresh fruits, after ISO 21527‐1:2008 [19]. DRBC (consisting of peptone 5 g/L; dextrose 10 g/L; potassium dihydrogen phosphate 1 g/L; magnesium sulphate 0.5 g/L; Rose Bengal 0.025 g/L; chloramphenicol 0.1 g/L; dichloran 0.002 g/L; agar 15 g/L) with an ending pH value of 5.6 ± 0.2 at 25°C is suggested for discriminating yeasts and fungi, specifically in foodstuff specimens (e.g., *Candida* spp., *Saccharomyces* spp., *Aspergillus* spp. and *Mucor* spp.). Afterwards, the cultivated dishes were kept at 25°C for 5 days. The results were expressed in logarithmic values of colony‐forming units per gram of the sample (log_10_ CFU/g).

TBC and TYMC were performed in accordance with the EC Regulations 2073/2005 and 1441/2007 [20 and 21].

### 2.7. Statistical Analysis

Each measurement was duplicated and reported as arithmetic average (±SD, *n* = 2). Statistical assessment has been made via GraphPad Prism Version 8.0.1 (GraphPad Software Inc., San Diego, California, United States). A two‐way ANOVA was used to analyse substantial modifications amongst A, *A* + Gly, *A* + RE and A + *G* *l* *y* + *R* *E* for each measurement, then Tukey′s multiple comparisons test was applied. Descriptive assessments has been made for all trials previous next tests. The Fmax test has been applied in order to analyse data homogeneity. Factor A has been symbolised by alginate, and adding glycerol or the extract represented Factor B. The same criteria were applied to the microbiological assessment of the fruits, where strawberries without film were considered Factor A. Statistical importance degrees have been given through the next symbols: *N.S. (not significant)* for *p* > 0.05 * *p* < 0.05, *** *p* < 0.001, ** *p* < 0.01.

## 3. Outcomes and Discussions

### 3.1. Polyphenols Amount Found in Aqueous RE

In this study, rosemary biomass, the residue after essential oil extraction, was repurposed via aqueous extraction to assess its polyphenolic content. The outcomes are expressed in Table 1 below, based on the MS and DAD spectra visible in Supporting Information attached (Figures S1–S14). It can be seen that up to 176.588 ± 3.83 mg/g polyphenols were obtained, with the major classes of polyphenols being hydroxybenzoic acids, hydroxycinnamic acids, flavones, flavonols, flavanones and phenolic terpenes. The three abundant compounds were rosmarinic acid (hydroxycinnamic acid, 60.353 ± 0.749 mg/g), luteolin‐glucuronide (flavone, 18.646 ± 0.28 mg/g) and isorhamnetin‐glucoside (flavonol, 12.577 ± 0.39 mg/g).

**Table 1:** DAD and MS data obtained after positive ionisation of the rosemary samples and the concentration of identified phenolic compounds, expressed as mg/g dry extract (±SD, *n* = 3).

Peak	R_t_	UV	[*M* + *H*]^+^	Phenolic compound	Subclass	Rosemary leaf extract
No.	(min)	*λ*_max_	(*m*/*z*)			
		(nm)				
1	3.62	280	139	Hydroxybenzoic acid	Hydroxybenzoic acid	0.733 ± 0.02
2	8.57	280	361	Syringic acid‐glucoside	Hydroxybenzoic acid	5.177 ± 0.30
3	12.21	280	139	*p*‐Hydroxybenzoic acid	Hydroxybenzoic acid	4.600 ± 0.01
4	13.65	290, 260	169	Vanilic acid	Hydroxybenzoic acid	1.129 ± 0.01
5	13.76	332	343	Caffeic acid‐glucoside	Hydroxycinnamic acid	1.970 ± 0.16
6	14.92	360, 260	465, 303	Quercetin‐glucoside	Flavonol	2.632 ± 0.08
7	16.14	280	199	Syringic acid	Hydroxybenzoic acid	9.969 ± 0.06
8	16.56	350, 260	449, 287	Kaempferol‐glucoside	Flavonol	5.416 ± 0.234
9	16.71	360, 270	479, 317	Isorhamnetin‐glucoside	Flavonol	12.577 ± 0.39
10	17.41	280	611, 303	Hesperidin	Flavanone	11.388 ± 0.28
11	18.27	330	361	Rosmarinic acid	Hydroxycinnamic acid	60.353 ± 0.749
12	18.85	340, 260	463, 287	Luteolin‐glucuronide	Flavone	18.646 ± 0.28
13	19.3	360, 270	493, 317	Isorhamnetin‐glucuronide	Flavonol	5.433 ± 0.33
14	19.96	330	339	Coumaroilquinic acid	Hydroxycinnamic acid	11.125 ± 0.12
15	20.85	360, 270	625, 317	Isorhamnetin‐rutinoside	Flavonol	6.210 ± 0.09
16	21.27	350, 260	287	Luteolin	Flavone	8.429 ± 0.26
17	23.18	330	347	Rosmanol	Phenolic terpene	5.483 ± 0.14
18	23.92	330, 290	331	Carnosol	Phenolic terpene	4.297 ± 0.22
19	25.83	290	333	Carnosic acid	Phenolic terpene	1.014 ± 0.04
				**Total phenolics**		176.588 ± 3.83

The aqueous RE has garnered significant attention in culinary and medicinal contexts due to its diverse bioactive components, aroma, flavour and health benefits. The identified phenolic compounds, particularly rosmarinic acid and related hydroxycinnamic derivatives, are widely recognised for their antioxidant and antimicrobial activities that are key contributors to the functional performance of the rosemary‐enriched seaweed‐derived coverings. Agreeing to the evidences of Blank et al. [22], the quantity of rosmarinic acid in aqueous RE can reach up to 153 mg/g, significantly higher than in its ethanol extract, which contains around 90 mg/g [22]. Additionally, in another investigation, it is mentioned that the phenolic content in rosemary can vary significantly, ranging from approximately 39.3 up to 523 mg/g across different plant‐derived extracts [23]. This variability may be driven by environmental factors, such as rosemary plants′ geographic origin and cultivation practises, which influence the metabolic pathways involved in phenolic biosynthesis [24, 25]. Moreover, Achour et al. identified different polyphenols in rosemary tea, highlighting bioactives like rosmarinic acid and other glycosides and hydroxybenzoates [26]. These findings point out the beneficial values of aqueous RE as an abundant base of antioxidants while offering a sustainable approach to repurposing spent biomass.

### 3.2. Film Solutions Characterisation

#### 3.2.1. Absorbance (600 nm), Transparency (nm/mm), Antioxidant Capacity (umol TE/100 g)

**Figure 1:**
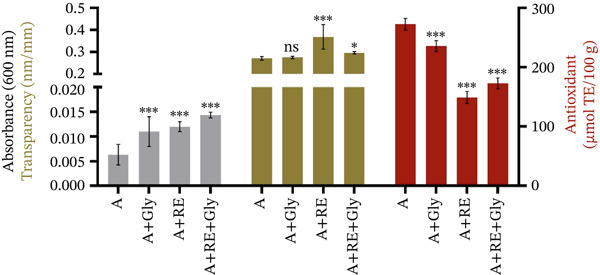
Light absorbance, transparency (opacity) and antioxidant capacity of alginate‐based solution enriched with aqueous RE and glycerol as a plasticiser (mean ± SD, *n* = 2). A—alginate, Gly—glycerol, RE—rosemary extract. Two‐way ANOVA: multiple comparisons: A versus *A* + RE, *A* + Gly, *A* + Gly + RE, *p* < 0.001***, *p* < 0.01**, *p* < 0.05*, *p* > 0.05^NS^.

Aqueous RE (9%) was incorporated into an alginate‐based matrix (2%). The solution was evaluated for its light absorbance, transparency degree and antioxidant capacity. Glycerol was supplemented as a plasticiser and consequently analysed. The results are expressed in Figure 1 and Table S1.

As shown in the figure above, the alginate coating shows lower values of light absorption and opacity. The addition of glycerol and vegetal extract of RE significantly influences these parameters (0.006 ± 0.002 nm vs. 0.011 ± 0.003^***^, 0.012 ± 0.001^***^ and 0.014 ± 0.001^***^ nm; 0.268 ± 0.006 nm/mm vs. 0.275 ± 0.006^NS^, 0.336 ± 0.114^***^ and 0.299 ± 0.012^*^; *p* < 0.001 ^***^, *p* < 0.033 ^*^, *p* > 0.123 > 0.123^NS^). UV radiation might trigger biochemical responses or degrade certain bioactive products that are prone to photodegradation. For that reason, light absorption capacity and film opacity are crucial factors in developing safe, biodegradable packaging materials, as they help protect bioactive compounds in food products from degradation while extending shelf life. Therefore, improving coating biopolymers, such as alginate, with other bioconstituents to prolong the lifespan of highly degradable items becomes increasingly necessary. Other studies support this statement, as the light absorption capacity and opacity of alginate‐based coatings can significantly influence the postharvest quality of fresh vegetal products. As for instance, Medina‐Jaramillo et al. [23] demonstrated that uncoated Andean blueberries appeared darker than those coated with alginate or alginate combined with carvacrol. This observation suggests that the evener area of covered horticultural products enhances the reflection of UV radiation, thereby improving aesthetic appeal [27]. Comparable effect has been reported in studies examining alginate coverings on the visual attributes of fruits, such as mango, where coatings improved aesthetic qualities [28]. Moreover, the light absorption characteristics of alginate‐based coatings can also play a critical role in protecting sensitive phytonutrients. Koh et al. showed that alginate coatings could effectively maintain carotenoid content in fresh‐cut cantaloupe by modifying the atmosphere within the coated fruit, thereby reducing exposure to light and oxygen that typically lead to degradation [29]. This indicates that alginate‐based coatings may serve as barriers to light and oxygen, diminishing the negative impact of external conditions on the nutritious quality of foods.

A reduction in antioxidant capacity was observed when examining the antioxidant properties of alginate‐based coverings enriched with rosemary bioactive compounds. The highest antioxidant values were found in alginate‐based matrices (A: 272.753 ± 9.961 and A + Gly: 235.762 ± 9.013^NS^). Incorporating the aqueous RE decreased this property, probably because of the interconnections between the alginate and polyphenolic compounds, leading to aggregate formation that may hinder their effectiveness in scavenging free radicals [30, 31]. One possible factor contributing to the decreased antioxidant capacity could be the concentration of bioactive compounds in the aqueous RE. Studies have shown that lower concentrations of rosmarinic acid and other phenolic compounds are associated with poorer antioxidant activity when incorporated into biopolymeric matrices [32].

#### 3.2.2. Viscosity Assessment

**Figure 2:**
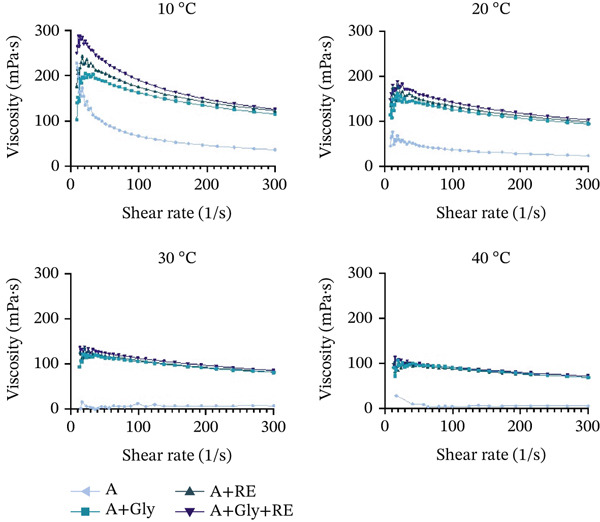
Rheological assessment in terms of viscosity at 10°C, 20°C, 30°C and 40°C for A—alginate, Gly—glycerol and RE—rosemary extract.

The viscosity of film solutions remains a very important parameter considering the visual aspect and covering layers′ effectiveness. Maintaining an ideal thickness is essential for achieving a uniform coating and forming a continuous, stable film stratum on the coating stretch [33]. Here, the rheological properties of seaweed‐derived coatings were analysed considering their flow behaviour. Results indicate that heat variations (alternating from 10°C to 40°C) precisely impact the alginate‐derived mixtures′ viscoelastic behaviour, as shown in Figure 2 and Tables S2.1–S2.4.

The rheological analysis of the film blends revealed that the seaweed‐derived formulations demonstrate shear‐thinning behaviour, with viscosity diminishing as the shear frequency increases. These characteristics benefit applications like covering, as the blend′s viscosity decreases beneath shear pressure, facilitating easier distribution and surface coverage [34]. Additionally, an increase in temperature was found to reduce the ability of alginate to form gels, making it more liquid and less firm (for example, 172.8 mPa?s at 10°C vs. Twenty eight.8 mPa?s at 40°C at a shear frequency of 17 s^−1^). Moreover, adding glycerol increases the fluids′ viscoelasticity at decreased temperatures (for example, 36.7 mPa?s vs. 115.1 mPa?s at 10°C and 300 s^−1^ shear frequency). This plasticiser can affect the heat susceptibility of alginate blends, influencing their gelation behaviour and viscoelastic characteristics across a range of temperatures. This effect is particularly relevant for uses involving heat fluctuations throughout manufacturing. In the current study, adding glycerol to seaweed‐derived mixture increased thickness on superior heat values (20°C–40°C) compared with inferior temperatures (10°C). The incorporation of plant essence, like RE, into seaweed‐derived formulations also affects their rheological attributes. Supplementation with RE significantly alters the flow behaviour of the alginate solution, making it more viscous across all shear rates, temperature ranges and glycerol contents (Tables S2.1–S2.4). This effect could have been associated with reactions between phenolic bioactive compounds and seaweed‐derived networks, which might influence solution′s stream behaviour. Additionally, the oxidation‐inhibiting elements in the extracts may act as crosslinking agents or chelators, contributing to the solution′s long‐term stability [35].

### 3.3. Firm Film Characterisation

#### 3.3.1. Physical Analyses

**Figure 3:**
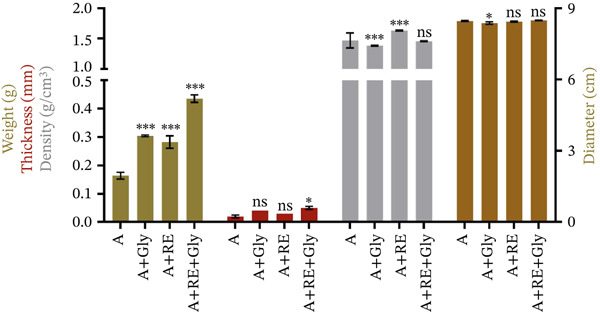
Physical measurements of alginate‐based solid biofilms (mean ± SD, *n* = 2). A—alginate, Gly‐glycerol and RE—rosemary extract. Significant differences were noted for *p* < 0.001^***^, *p* < 0.002^**^, *p* < 0.05^*^ and ns for *p* > 0.05^NS^.

The values for the firm coverings physical assessments are presented in Figure 3 and Table S3.

Regarding appearance, compact coatings supplemented with glycerol exhibited noticeable modifications in the softness, plasticity, stretching capacity, uniformity and homogeneousness of the surface. The addition of plasticiser was found to reduce inner resistance of the polymer network, leading to an evener, extra uniform area [36]. In addition, the decreased brittleness leads to an extra malleable, less unbending coating material, making it a more suitable product for diverse packing approaches. The improved plasticity enables the film to adapt to asymmetrical contours and sides, enhancing its adaptability for various wrapping and coating uses [35, 36].

The supplementation with aqueous RE also affected the physical characteristics of the solid films. Alginate‐based control films exhibited significantly lower values (*** *p* < 0.001) for weightiness, width and compactness, excluding the breadth, where no notable modifications were observed. Furthermore, the RE influenced the films′ texture and surface properties.

These physical measurements directly influence the performance of alginate as an obstacle towards external factors, like humidity and oxygen, which are amongst the greatest quality parameters concerning the foodstuffs life extension throughout storing. For instance, the thickness of alginate coatings is an essential parameter; studies have shown that it can be controlled by the viscidness of the covering formulation and the application rate. Specifically, it has been shown that as the coating solution′s viscosity increases, the final film′s thickness also increases proportionally [3, 4]. Furthermore, application methods such as vacuum impregnation can yield more uniform coatings than simple dipping, potentially affecting the efficacy of coatings on food items [37]. In addition, significant differences in film thickness were reported, impacting the overall weight gain of the coated melon, indicating that optimised thickness directly correlates with the preservation capabilities of the coating [3, 4, 37].

Likewise, alginate‐based coatings′ weight represents a very important aspect in food preservation. Lighter coatings may allow better adhesion and less impact on the food′s textural quality, whereas heavier coatings can provide more substantial barriers but may compromise sensory properties [28]. Regarding density, alginate‐based films often exhibit a notable variation depending on the incorporation of additional components within the coating matrix, such as RE. The density of alginate films is essential for understanding their film‐forming abilities and interactions with other nutrients or active substances. For example, incorporating agents such as rosmarinic acid, carvacrol, aqueous extracts, or essential oils into the alginate matrix may alter the density and, consequently, influence the blockade characteristics of the covering material, providing benefits beyond mere physical protection [2, 38].

#### 3.3.2. Moistness Amount, WST, WVP

**Figure 4:**
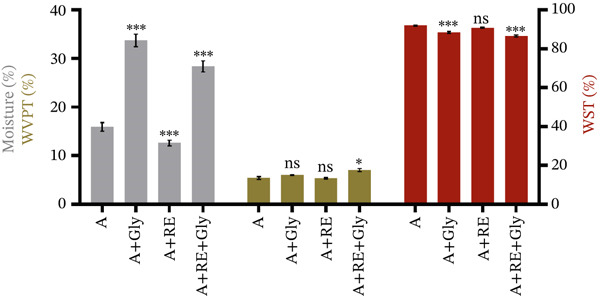
Moisture content, water solubility test (WST) and water vapour permeability (WVP) of the alginate‐based solid coatings (mean ± SD, *n* = 2). A—alginate, Gly—glycerol and RE—rosemary extract. Significant differences were noted for *p* < 0.001^***^, *p* < 0.01^**^, *p* < 0.05^*^, *p* > 0.05^NS^.

Figure 4 and Table S4 illustrate the moisture content, WST and WVP results.

As can be observed, in seaweed‐derived formulations, the supplementation of RE significantly decreased the moisture content (A + RE, *** *p* < 0.01). Additionally, the presence of the plasticiser strongly influenced water retention within the alginate matrix, regardless of RE supplementation (A + Gly, A + RE + Gly, *** *p* < 0.01). However, in dissolution tests, films with glycerol addition exhibited lower water solubility (A + Gly, A + RE + Gly, *** *p* < 0.01). Higher WST values were observed in alginate‐based films containing RE without glycerol, with no substantial modifications from the blank samples. Even though glycerol is a hydrophilic compound, it reduces film dissolution and improves firmness [36]. The outcome might be connected to glycerol′s property of modifying hydrophilic‐hydrophobic balance of the film matrix, enhancing hydrophobic interactions and reducing coating’s attraction to H_2_O, thereby restraining its dissolution [39]. Conversely, the WVP test of seaweed‐derived coatings remained slightly modified when glycerol was absent and RE incorporated. In addition, when glycerol was added, WVP increased slightly in both alginate‐based films with and without RE supplementation. This effect may result from interactions between glycerol, RE polyphenols at different concentrations, and the alginate matrix. Moreover, processing conditions such as heat, dehydrating techniques and curing period could lead to structural changes that increase pore size and enhance WVP.

Studies indicate that alginate coatings can significantly reduce weight loss in coated fruits during storage, reflecting the coating′s effectiveness in maintaining moisture levels [2]. Specific weights measured in detailed studies have shown consistent performance in retaining the physical quality of foods, particularly in preventing excessive dehydration of fruits such as strawberries and other products [28]. In addition, research studies have noted that alginate films typically exhibit a high degree of hydrophilicity, which, combined with their density, determines the coatings′ moisture retention [40, 41].

#### 3.3.3. Microbiological Assessment of Fresh Fruits Coated With Film Solution

**Figure 5:**
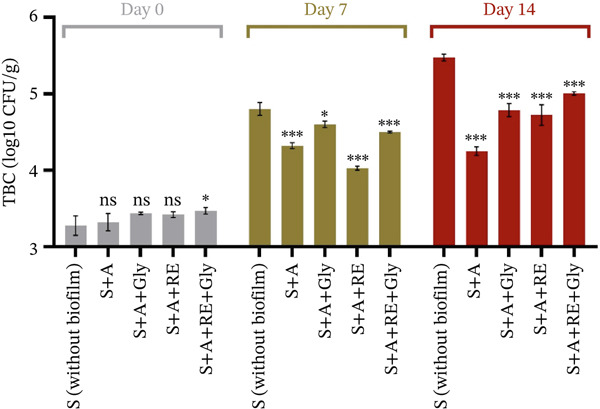
Total bacteria count (TBC), log10 CFU/g. S‐uncoated strawberry control, A—alginate, Gly—glycerol and RE—rosemary extract. Significant differences were noted for *p* < 0.001^***^, *p* < 0.01^**^, *p* < 0.05^*^, *p* > 0.05^NS^.

**Figure 6:**
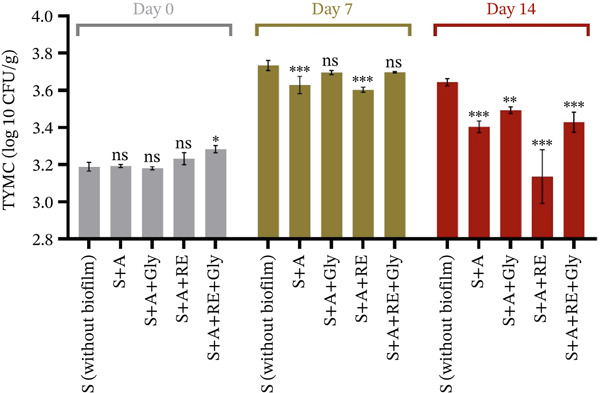
Total yeasts and molds count (TYMC), log10 CFU/g. S‐uncoated strawberry control, A—alginate, Gly—glycerol and RE—rosemary extract. Significant differences were noted for *p* < 0.001^***^, *p* < 0.01^**^, *p* < 0.05^*^, *p* > 0.05^NS^.

In this study, alginate‐based films enriched with RE were tested for their antimicrobial potential by coating highly perishable foods, such as strawberries. The fruits were tested with and without coating at the start of the trial, after 7 and 14 days of storage. TBC and TYMC were performed, and the outcomes are shown in Figure 5 (plus Table S5) and Figure 6 (plus Table S6).

As shown in Figure 5 and Table S5, at the start of the trial (Day 0), the total bacterial load in fruits covered with the alginate‐based solution was similar to that without the film (ranging from 3.421 ± 0.127 to 3.484 ± 0.044 log10 CFU/g). The situation changes with storage at 1 and 2 weeks, as significant differences emerge between uncoated fruits and those covered with an alginate‐based film. After 7 days of storage, the strawberries covered with alginate enriched with RE showed the lowest TBC (4.052 ± 0.026 vs. 4.733 ± 0.083 log10 CFU/g in strawberries without film, *p* < 0.001 ***). After 14 days maintained under refrigerated conditions, the strawberries covered with alginate and those enriched with RE showed the lowest TBC (4.243 ± 0.055 and 4.871 ± 0.135 log10 CFU/g, *p* < 0.001 ***). The addition of glycerol to the alginate‐based film slightly influenced TBC at 7 days of storing (*p* < 0.05*) compared with the control, but significantly influenced the values when RE was supplemented (*p* < 0.001 ***). Moreover, after 14 days of storage, trials containing glycerol showed higher TBC values than those without glycerol but significantly lower than the control (strawberries without film, *p* < 0.001 ***).

Strawberries, as a perishable fruit, are particularly susceptible to microbial spoilage; thus, various methods are employed to prolong their lifespan, including the use of comestible coverings, such as sodium alginate, and the incorporation of natural extracts, such as rosemary. Researchers reported that alginate‐derived coverings might efficiently retain moisture and decrease respiration rates in strawberries, thereby slowing down decay [37, 42]. Moreover, incorporating bioactive compounds, such as polyphenols from RE, into alginate coatings further enhances this protective effect. RE exerts antimicrobial activity primarily through its polyphenols—like rosmarinic and carnosic acids, and carnosol—which break the external membranes of bacterial cells, intensify the penetrability of membranes, and last but not the least, obstruct metabolic and enzymatic functions. These mechanisms collectively reduce bacterial growth on the fruit surface. In addition, studies show that alginate coatings enriched with olive leaf extract have demonstrated significant efficacy in retaining phenolic compounds in cherries, suggesting a similar potential for strawberries [43]. This is critical as polyphenols are recognised for their positive effect against microorganisms, which may help obstruct the development of spoilage strains on the external side of fresh fruits [44, 45]. Furthermore, the RE potential against microbial growth in strawberry coatings has been highlighted, supporting its role in further reducing bacterial loads during storage, as it possesses inherent antibacterial properties [46, 47]. This can significantly contribute to maintaining strawberries′ overall quality and safety, particularly when combined with alginate coatings, which provide an additional protective layer. Implementing such coatings may thus enhance the strawberry viability during postharvest handling and marketing [48, 49]. Additionally, it should be noted that edible coatings are designed primarily to delay microbial proliferation rather than to achieve complete microbial inactivation, and therefore, even moderate reductions in growth rates represent meaningful improvements in shelf‐life preservation.

Figure 6 and Table S6 illustrate the TYMC situation in uncoated strawberries and in strawberries covered with an alginate‐based film.

As in the case of bacterial growth, the supplementation of RE to the alginate‐based formulation significantly lowered the TYMC after 7 (from 3.734 ± 0.027 to 3.602 ± 0.013 log10 CFU/g, *p* < 0.001 ***) and 14 days of storage (from 3.643 ± 0.019 to 3.135 ± 0.144 log10 CFU/g, *p* < 0.001 ***). The glycerol addition gave no significant reduction of TYMC after 7 days, only after 14 days of storage (3.428 ± 0.053 log10 CFU/g, *p* < 0.001 ***). This substantial decrease in TYMC after 2 weeks, but not after just 1 week, can be attributed to the prolonged antimicrobial action of the alginate‐glycerol‐RE mixture. Initially, the bioactive compounds in RE, such as hydroxycinnamic acids and flavonols, may take time to exert their full antifungal effects, especially as microbial populations adapt to environmental stressors. Additionally, the coating may have gradually modified the fruit′s surface environment, reducing the availability of moisture and inhibiting oxygen diffusion, thereby delaying fungal growth over time. Last but not least, the observed antimicrobial activity likely arises from the controlled distribution of polyphenols from the alginate blend to the fruit surface, creating a localised antimicrobial environment.

Anyhow, our results are supported by the scientific literature. Research has shown that strawberries coated with sodium alginate exhibit less weight loss and a lower microbial load than uncoated fruits when stored under the same conditions. As an example, the weightiness decrease of strawberries covered in a sodium alginate‐calcium chloride mixture was considerably inferior to the control, which experienced visible mould growth after 1 week of storage [50]. This demonstrates that alginate‐based coatings can be a barrier to moisture loss and a physical deterrent to microbial invasion [51]. Moreover, incorporating vegetal extracts, such as RE, into alginate coatings can further enhance their antifungal properties. Rosemary has been noted for its ability to combat a variety of molds, significantly reducing yeast and mould counts [52]. An investigation of the effects of various edible coatings, including rosemary‐coated coatings, reported a marked reduction in TYMC compared with control groups, thereby supporting the potential of alginate and RE combinations to extend the lifespan of strawberries by effectively monitoring microbial growth [53]. Moreover, applying seaweed‐derived coverings, especially when enriched with natural essences such as rosemary, presents a viable strategy for reducing total microbial loads in highly perishable fruits like strawberries. This enhances the fruits′ shelf life and ensures a higher‐quality product for consumers [54, 55 and 56]. Nonetheless, selecting strawberries as a model fruit provides a scientifically valid and industrially relevant platform for testing film functionality, given their high perishability and susceptibility to oxidative stress. The demonstrated preservation effects thus offer strong translational potential for other soft fruits with similar physiology. Although scalability testing was beyond the scope of this initial study, the formulation components—sodium alginate and RE—are low‐cost, food‐grade and compatible with existing edible coating lines, supporting straightforward industrial translation.

This study advances the search for sustainable coating solutions for highly perishable fruits in response to the growing demand for eco‐friendly alternatives. Although a key objective of this study was to valorise the biomass by‐product from rosemary distillation by integrating its bioactive compounds into alginate‐based films, certain limitations must be considered. The research primarily examines antioxidant properties, film characteristics and their effects on perishable fruit shelf life, yet challenges such as scalability, cost‐efficiency and interactions influencing antioxidant activity remain. The microbiological quality of the fresh fruits coated with alginate solutions was monitored over only 2 weeks, suggesting that longer‐term studies are necessary to fully understand the preservation efficacy over extended periods. Although this study focused on strawberries, it does not cover a wide range of perishable items, limiting the applicability of its findings to other food products. Additionally, processing conditions (e.g., heat, aeriation and curing period) may influence film penetrability and performance, highlighting the need for further optimisation. Last but not the least, the study primarily examines specific film properties and may not account for all probable parameters or real‐life conditions that might negatively influence the concrete implementation and scale‐up potential of the proposed ecological covering alternatives.

## 4. Conclusions

Our research investigated the capacity of alginate‐based films enriched with RE as sustainable bio‐coatings. Rich in polyphenolic compounds, RE contributed to enhanced packaging properties and improved food preservation. Its incorporation influenced the film′s antioxidant capacity, UV–Vis light protection and rheological behaviour. Additionally, the RE affected moisture retention, dissolution in water and water vapour penetrability. Whereas glycerol reduced solubility, it raised vapour permeability, highlighting the complex interactions between components and their impact on the film′s functional properties. Moreover, this study found that strawberries coated with RE‐enriched alginate significantly reduced TBC compared with uncoated fruits. Similarly, the inclusion of RE significantly reduced TYMC in strawberries stored for 14 days at 5°C. Overall, adding RE to alginate coatings plays a crucial role in diminishing microscopic content on fresh strawberries, thereby improving their lifespan and superior features while storing.

This study provides valuable insights into sustainable packaging solutions, addressing the increasing need for sustainable replacements across industries. Future research should focus on refining preparation and manufacturing techniques to overcome bottlenecks like moisture content, water vapour penetrability, or microbiological load, ultimately improving performance and growing the possible uses of these ecological materials in sustainable packaging for highly perishable food items.

## Author Contributions

**Laura Mitrea:** writing – original draft, conceptualization. **Bernadette-Emöke Teleky:** writing – review & editing, investigation. **Silvia-Amalia Nemeş:** investigation, formal analysis. **Mihaela-Ştefana Păşcuţă:** formal analysis. **Adrian-Gheorghe Martău:** methodology, data curation. **Lavinia-Florina Călinoiu:** writing – review & editing, validation, supervision. **Alina-Lăcrămioara Nistor:** investigation. **Carmen-Rodica Pop:** writing – review & editing. **Ancuţa-Mihaela Rotar:** writing – review & editing. **Francisc-Vasile Dulf:** formal analysis. **Bianca-Eugenia Ştefănescu:** investigation. **Magdalini Krokida:** validation, resources. **Dan-Cristian Vodnar:** project administration, funding acquisition.

## Funding

This research was funded by the European Union′s Horizon 2020 research and innovation programme under the Marie Skłodowska‐Curie Grant Agreement (No. 101007783‐FRIETS) and Unitatea Executiva pentru Finantarea Invatamantului Superior, a Cercetarii, Dezvoltarii si Inovarii România (PN‐IV‐P1‐PCE‐2023‐1092, PN‐IV‐P1‐PCE‐2023‐1092).

## Disclosure

The authors take full responsibility for the integrity, originality and content of the manuscript.

## Ethics Statement

The authors have nothing to report.

## Conflicts of Interest

The authors declare no conflicts of interest.

## Supporting Information

Additional supporting information can be found online in the Supporting Information section.

## Supporting information


**Supporting Information 1** Figures S1–S14: Mass spectrometry (MS) spectra and diode‐array detector (DAD) chromatograms corresponding to the identification and characterization of major compounds in rosemary extract (quercetin‐glucoside, kaempferol‐glucoside, isorhamnetin‐glucoside, hesperidin, rosmarinic acid, luteolin‐glucuronide), along with the DAD chromatograms for the whole rosemary extract at 280 and 340 nm.

**Supporting Information 2** Tables S1–S6: Physicochemical, optical, functional and microbiological characterization of alginate‐based films, including: visual appearance; absorbance at 600 nm; transparency (nm/mm); antioxidant activity (*μ*mol TE/100 g); rheological measurements conducted at 10°C, 20°C, 30°C and 40°C; physical parameters (weight, thickness, diameter, density); moisture content; water solubility; water vapour permeability; and microbial analysis (total bacterial load, total yeast and mould counts expressed as CFU/g).

## Data Availability

Data are available on request from the authors.
