# Supplementary material for: Functional Characterisation of Rosemary‐Enhanced Alginate Films for Strawberry Preservation
Source: Int J Food Sci. 2026 May 21;2026:5597893. doi: 10.1155/ijfo/5597893 (PMC13191819; doi:10.1155/ijfo/5597893)
Supplement: Supplementary file 1 — Supporting Information 1 Figures S1–S14: Mass spectrometry (MS) spectra and diode‐array detector (DAD) chromatograms corresponding to the identification and characterization of major compounds in rosemary extract (quercetin‐glucoside, kaempferol‐glucoside, isorhamnetin‐glucoside, hesperidin, rosmarinic acid, luteolin‐glucuronide), along with the DAD chromatograms for the whole rosemary extract at 280 and 340 nm. [file IJFO-2026-5597893-s002.doc]

Figure S1. MS spectra of quercetin-glucoside (Rt=14.92 [M+H] = 465, 303)

Figure S2. DAD chromatogram for quercetin-glucoside

Figure S3. MS spectra of kaempferol-glucoside (Rt=16.56 min [M+H] = 449, 287)

Figure S4. DAD chromatogram of kaempferol-glucoside

Figure S5. MS spectra of isorhamnetin-glucoside (Rt=16.71 [M+H] = 479, 317)

Figure S6. DAD chromatogram of isorhamnetin-glucoside

Figure S7. MS spectra of hesperidin (Rt=17.41 min [M+H] = 611)

Figure S8. DAD chromatogram of hesperidin

Figure S9. MS spectra of rosmarinic acid (Rt=18.27 min [M+H] = 361, 163)

Figure S10. Chromatograms of rosmarinic acid

Figure S11. MS spectra of luteolin-glucuronide (Rt=18.85 [M+H] = 463)

Figure S12. Chromatograms of luteolin-glucuronide

Figure S13. DAD chromatograms of rosemary leaf extract (280 nm)

Figure S14. DAD chromatograms of rosemary leaf extract (340 nm)
