# Supplementary material for: Functional Characterisation of Rosemary‐Enhanced Alginate Films for Strawberry Preservation
Source: Int J Food Sci. 2026 May 21;2026:5597893. doi: 10.1155/ijfo/5597893 (PMC13191819; doi:10.1155/ijfo/5597893)
Supplement: Supplementary file 2 — Supporting Information 2 Tables S1–S6: Physicochemical, optical, functional and microbiological characterization of alginate‐based films, including: visual appearance; absorbance at 600 nm; transparency (nm/mm); antioxidant activity (μmol TE/100 g); rheological measurements conducted at 10°C, 20°C, 30°C and 40°C; physical parameters (weight, thickness, diameter, density); moisture content; water solubility; water vapour permeability; and microbial analysis (total bacterial load, total yeast and mould counts expressed as CFU/g). [file IJFO-2026-5597893-s001.docx]

**Table S1. Appearance, Absorbance (600 nm), Transparency (nm/mm), Antioxidant activity (umol TE/100g)**

| **Sample, Appearance** | **Absorbance (nm)** | **Transparency (nm/mm)** | **Antioxidant (umol TE/100mg)** |
| --- | --- | --- | --- |
| 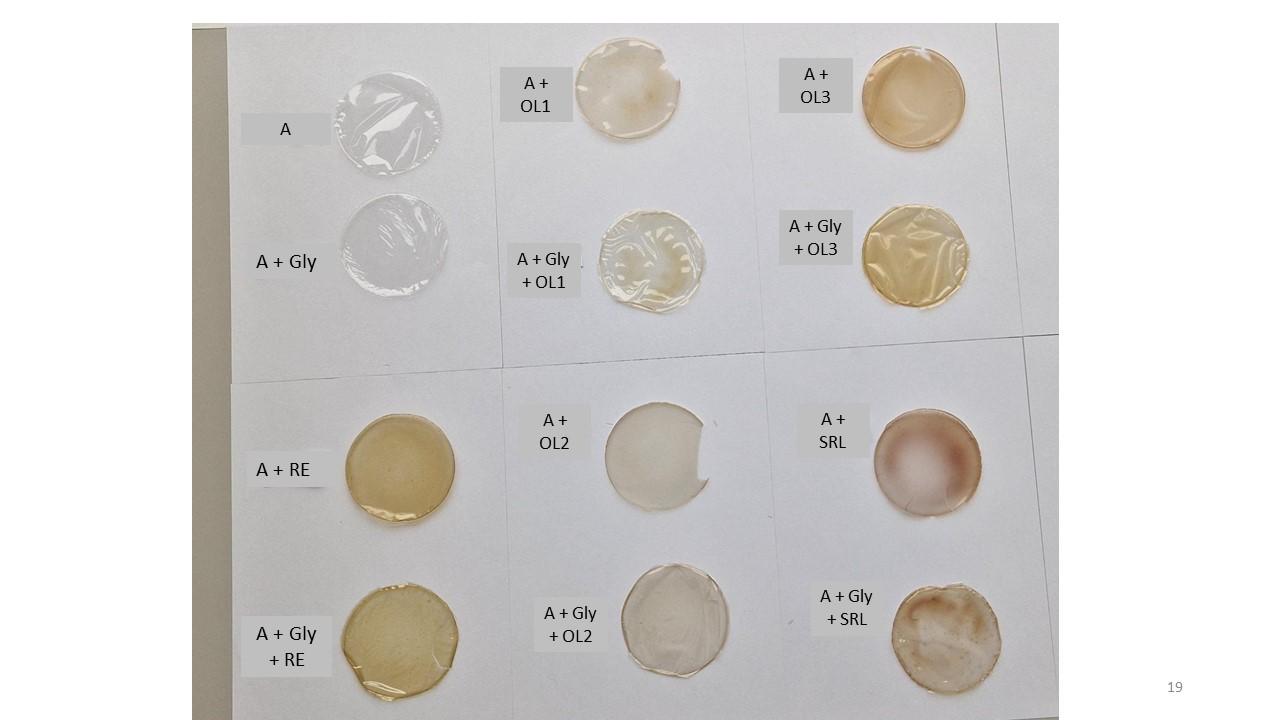**A** | 0.006 ± 0.002 | 0.268 ± 0.006 | 272.753 ± 9.961 |
| 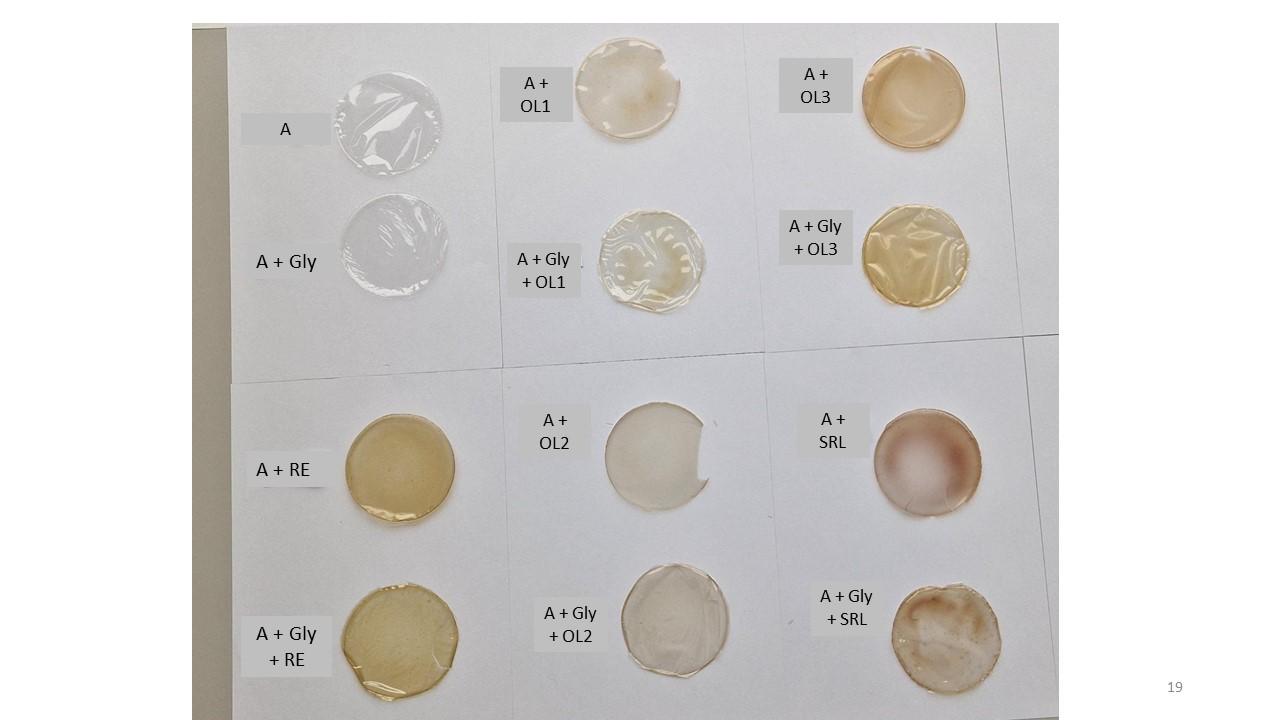**A+Gly** | 0.011 ± 0.003^***^ | 0.275 ± 0.006^NS^ | 235.762 ± 9.013 ^NS^ |
| 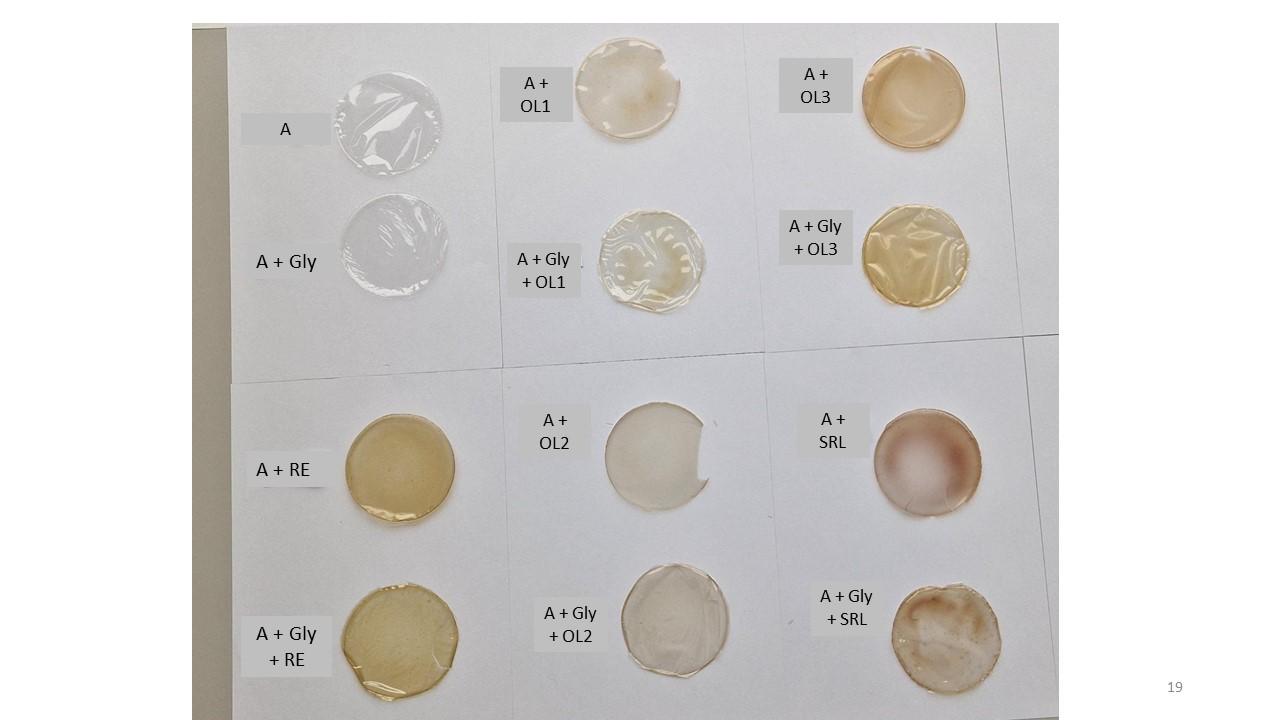**A+RE** | 0.012 ± 0.001^***^ | 0.336 ± 0.114 ^***^ | 149.227 ± 10.059 ^***^ |
| 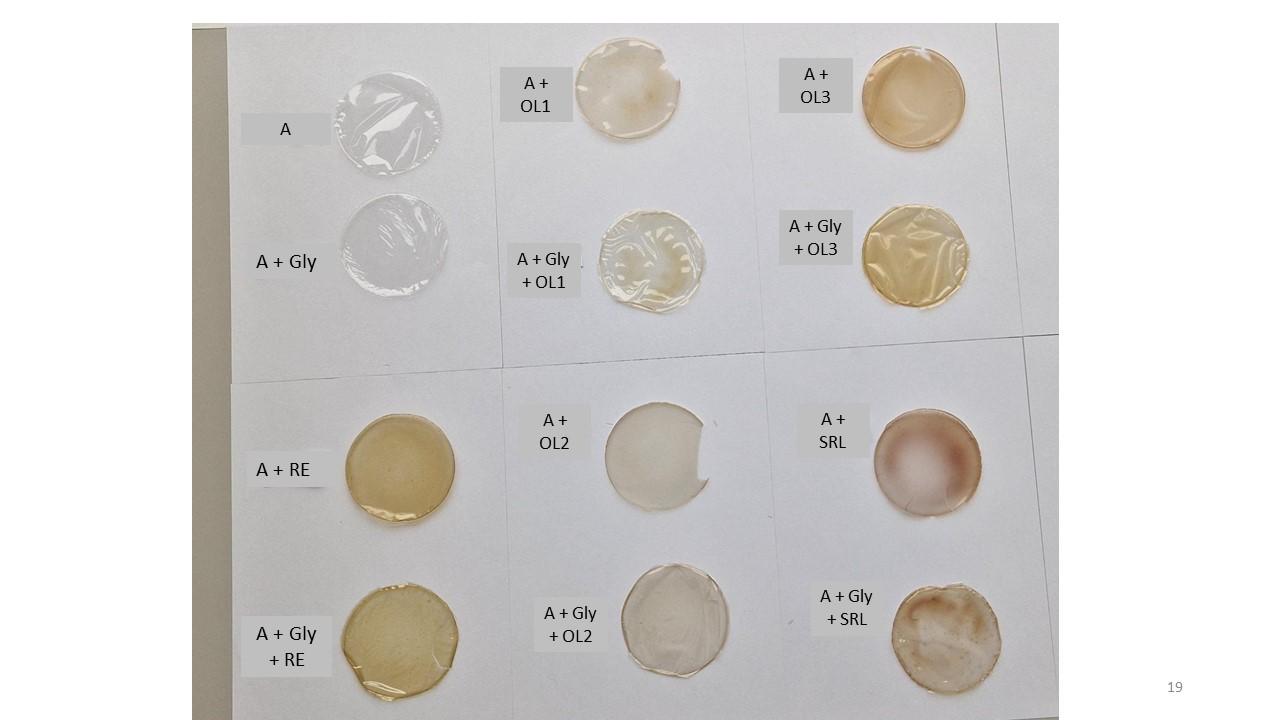**A+RE+Gly** | 0.014 ± 0.001^***^ | 0.299 ± 0.012 ^*^ | 173.043 ± 9.191^***^ |

*Two-way ANOVA: multiple comparison*

*A vs. A+RE, A+Gly, A+Gly+RE*

*p<0.001 ***, p<0.01**, p<0.05*, p>0.05 ^NS^*

**Tables S2. Rheological assessment**

***Table S2.1. At 10℃***

| **10℃** | **Shear Rate** | **A** | **A+Gly** | **A+RE** | **A+Gly+RE** |
| --- | --- | --- | --- | --- | --- |
|  | **[1/s]** | **[mPa·s]** | **[mPa·s]** | **[mPa·s]** | **[mPa·s]** |
|  |  |  |  |  |  |
|  | 5.58 | 269.4 | 26.5 | 105 | 301.4 |
|  | 6.23 | 247.4 | 61 | 146 | 290.9 |
|  | 6.97 | 226.7 | 77.9 | 171.8 | 305.1 |
|  | 7.78 | 211.8 | 93.5 | 156 | 282.4 |
|  | 8.7 | 227.4 | 103.2 | 176.3 | 249.3 |
|  | 9.71 | 217.4 | 140.2 | 213.8 | 263 |
|  | 10.9 | 199.2 | 152.2 | 206.2 | 286.8 |
|  | 12.1 | 163.6 | 144.3 | 194 | 264 |
|  | 13.5 | 165.7 | 191.2 | 217.1 | 287.9 |
|  | 15.1 | 154.6 | 183.4 | 229.4 | 279.4 |
|  | 16.9 | 172.8 | 197.1 | 242.8 | 284.2 |
|  | 18.9 | 154.7 | 194.9 | 225.8 | 269.5 |
|  | 21.1 | 134.8 | 205.2 | 227 | 275.9 |
|  | 23.5 | 143.4 | 195.3 | 236 | 269 |
|  | 26.3 | 125.4 | 201.8 | 221.9 | 262.4 |
|  | 29.4 | 127.9 | 198.2 | 227.4 | 255.6 |
|  | 32.8 | 112.9 | 203.4 | 220.4 | 254.1 |
|  | 36.6 | 107.9 | 194 | 212.2 | 245.2 |
|  | 40.9 | 104.3 | 191.8 | 211 | 239.5 |
|  | 45.7 | 99.3 | 188.6 | 207.2 | 234.9 |
|  | 51.1 | 93.5 | 186.9 | 205 | 230 |
|  | 57.1 | 89.6 | 182.1 | 200 | 222.7 |
|  | 63.7 | 85 | 178.8 | 195.7 | 217.6 |
|  | 71.2 | 79.8 | 175.2 | 191.2 | 210.8 |
|  | 79.5 | 75.5 | 171 | 185.8 | 204.4 |
|  | 88.8 | 71.1 | 166.8 | 180.2 | 197.3 |
|  | 99.2 | 67.1 | 162.7 | 175.4 | 190.7 |
|  | 111 | 63.4 | 158.3 | 170 | 183.3 |
|  | 124 | 59.8 | 153.7 | 164.8 | 177.2 |
|  | 138 | 56.5 | 149.1 | 159.1 | 169.7 |
|  | 154 | 53.2 | 144.3 | 154.1 | 163 |
|  | 173 | 50.2 | 139.4 | 148.6 | 156.4 |
|  | 193 | 47.2 | 134.6 | 143.2 | 149.9 |
|  | 215 | 44.4 | 129.9 | 137.9 | 143.6 |
|  | 240 | 41.8 | 125 | 132.7 | 137.5 |
|  | 269 | 39.2 | 120.1 | 127.6 | 131.5 |
|  | 300 | 36.7 | 115.1 | 122.3 | 125.4 |

***Table S2.2. At 20℃***

| **20℃** | **Shear Rate** | **A** | **A+Gly** | **A+RE** | **A+Gly+RE** |
| --- | --- | --- | --- | --- | --- |
|  | **[1/s]** | **[mPa·s]** | **[mPa·s]** | **[mPa·s]** | **[mPa·s]** |
|  |  |  |  |  |  |
|  | 8.7 | 45.6 | 114.5 | 117.8 | 147.4 |
|  | 9.71 | 64 | 137.7 | 135.1 | 160.8 |
|  | 10.9 | 67.9 | 108 | 145.3 | 158.9 |
|  | 12.1 | 77.4 | 148.3 | 175.6 | 179.6 |
|  | 13.5 | 49.4 | 124.2 | 161.1 | 172.9 |
|  | 15.1 | 60.3 | 137.2 | 148.1 | 167.1 |
|  | 16.9 | 56.5 | 153.6 | 147 | 171.8 |
|  | 18.9 | 68.5 | 161.9 | 178.1 | 188.3 |
|  | 21.1 | 59.6 | 146.8 | 175 | 178.2 |
|  | 23.5 | 55.6 | 155.6 | 158.4 | 176.6 |
|  | 26.3 | 59.2 | 144.2 | 167.1 | 182 |
|  | 29.4 | 54.6 | 150.4 | 156.1 | 171.4 |
|  | 32.8 | 50.1 | 143 | 158.3 | 170.8 |
|  | 36.6 | 54.6 | 144.8 | 161.7 | 170.2 |
|  | 40.9 | 51.6 | 146.1 | 156.4 | 168.1 |
|  | 45.7 | 49.3 | 142.2 | 153.6 | 164.6 |
|  | 51.1 | 45.8 | 141.2 | 149.8 | 159.6 |
|  | 57.1 | 45.2 | 139.4 | 147.7 | 158.5 |
|  | 63.7 | 43.3 | 137.1 | 144.7 | 155.4 |
|  | 71.2 | 41.9 | 134.4 | 142.2 | 152.6 |
|  | 79.5 | 40.8 | 132.6 | 140.1 | 149.6 |
|  | 88.8 | 38.9 | 129.6 | 137.4 | 146.7 |
|  | 99.2 | 37.4 | 126.6 | 134.5 | 142.5 |
|  | 111 | 36 | 123.8 | 131.4 | 138.9 |
|  | 124 | 34.8 | 121.1 | 128.1 | 135.3 |
|  | 138 | 33.1 | 118.1 | 124.5 | 131.6 |
|  | 154 | 31.6 | 115 | 120.7 | 127.6 |
|  | 173 | 32 | 111.8 | 117.1 | 123.7 |
|  | 193 | 28.9 | 108.5 | 113.9 | 119.7 |
|  | 215 | 27.9 | 105.1 | 110.3 | 115.5 |
|  | 240 | 26.7 | 101.9 | 106.4 | 111.6 |
|  | 269 | 25.6 | 98.4 | 102.9 | 107.7 |
|  | 300 | 24.1 | 94.8 | 99.1 | 103.5 |

***Table S2.3. At 30℃***

| **30℃** | **Shear Rate** | **A** | **A+Gly** | **A+RE** | **A+Gly+RE** |
| --- | --- | --- | --- | --- | --- |
|  | **[1/s]** | **[mPa·s]** | **[mPa·s]** | **[mPa·s]** | **[mPa·s]** |
|  |  |  |  |  |  |
|  | 13.5 |  | 93.8 | 125.9 | 136.7 |
|  | 15.1 | 1.5 | 107.3 | 122.4 | 129.9 |
|  | 16.9 | 16 | 104.6 | 113.4 | 132.5 |
|  | 18.9 |  | 122.2 | 114.4 | 127.9 |
|  | 21.1 |  | 120.3 | 135.6 | 138.1 |
|  | 23.5 | 6.7 | 113.8 | 123.2 | 124.6 |
|  | 26.3 |  | 118.7 | 127.9 | 134.1 |
|  | 29.4 | 4 | 117.7 | 116.3 | 124.5 |
|  | 32.8 | 3 | 115.7 | 123.6 | 131.8 |
|  | 36.6 | 0.2 | 120.3 | 122.1 | 128.1 |
|  | 40.9 | 2.5 | 118 | 119.7 | 126.4 |
|  | 45.7 | 6.4 | 115.6 | 119.5 | 126.1 |
|  | 51.1 | 5.4 | 113.6 | 118.5 | 124.6 |
|  | 57.1 | 3.4 | 113.5 | 115.7 | 122.3 |
|  | 63.7 | 7.4 | 111.6 | 115.3 | 121.3 |
|  | 71.2 | 6.7 | 110.5 | 114.3 | 119.5 |
|  | 79.5 | 5.5 | 109.3 | 112.5 | 117.4 |
|  | 88.8 | 7.1 | 107.6 | 110.6 | 115.5 |
|  | 99.2 | 13 | 105.9 | 108.5 | 113.2 |
|  | 111 | 6.1 | 103.8 | 105.9 | 111.1 |
|  | 124 | 10.4 | 101.8 | 103.5 | 108.6 |
|  | 138 | 6.8 | 99.6 | 101.1 | 106.2 |
|  | 154 | 7.3 | 97.1 | 98.6 | 103.5 |
|  | 173 | 7.4 | 94.7 | 96 | 100.8 |
|  | 193 | 7.5 | 92.1 | 93.3 | 98 |
|  | 215 | 7.5 | 89.4 | 90.8 | 95 |
|  | 240 | 7.6 | 86.6 | 88.5 | 92 |
|  | 269 | 8.1 | 84.3 | 85.4 | 89 |
|  | 300 | 7.7 | 81.6 | 82.4 | 85.9 |

***Table S2.4. At 40℃***

| **40℃** | **Shear Rate** | **A** | **A+Gly** | **A+RE** | **A+Gly+RE** |
| --- | --- | --- | --- | --- | --- |
|  | **[1/s]** | **[mPa·s]** | **[mPa·s]** | **[mPa·s]** | **[mPa·s]** |
|  |  |  |  |  |  |
|  |  |  | 91.5 | 102.1 | 96.9 |
|  |  |  | 72.4 | 83 | 113.6 |
|  | 16.9 | 28.8 | 86.6 | 92.4 | 83.5 |
|  | 18.9 |  | 107.5 | 85.3 | 94.5 |
|  | 21.1 |  | 96.3 | 101.8 | 109.4 |
|  | 23.5 |  | 97 | 90.1 | 106.9 |
|  | 26.3 |  | 101.2 | 99.9 | 99.8 |
|  | 29.4 |  | 92.6 | 95.2 | 100.1 |
|  | 32.8 |  | 95.1 | 101.7 | 105.7 |
|  | 36.6 |  | 98 | 96.8 | 99.3 |
|  | 40.9 | 10.7 | 100.4 | 95 | 96.6 |
|  | 45.7 |  | 98.4 | 96.3 | 98.4 |
|  | 51.1 |  | 95.4 | 95.7 | 100.1 |
|  | 57.1 | 9.8 | 96.7 | 93.9 | 96.6 |
|  | 63.7 | 4 | 95.8 | 93.2 | 96 |
|  | 71.2 |  | 94.5 | 92.9 | 95.7 |
|  | 79.5 | 5.2 | 94.5 | 90.9 | 94.1 |
|  | 88.8 | 5.6 | 93.2 | 90.2 | 92.9 |
|  | 99.2 | 5 | 91.5 | 89 | 91.8 |
|  | 111 |  | 89.4 | 87.4 | 90.4 |
|  | 124 | 5.5 | 87.6 | 85.1 | 88.8 |
|  | 138 | 7.8 | 85.7 | 83.5 | 87.2 |
|  | 154 | 5.9 | 83.6 | 81.7 | 85.3 |
|  | 173 | 6.1 | 81.9 | 79.9 | 83.4 |
|  | 193 | 6.2 | 79.6 | 78.2 | 81.3 |
|  | 215 | 6.3 | 77.2 | 75.7 | 79.1 |
|  | 240 | 6.3 | 73.9 | 73.9 | 77 |
|  | 269 | 6.4 | 71.8 | 71.9 | 74.7 |
|  | 300 | 6.3 | 69.4 | 69.6 | 72.4 |

**Table S3. Physical measurements**

|  | **A** | **A+RE** | **A+Gly** | **A+Gly+RE** |
| --- | --- | --- | --- | --- |
| **Weight (g)** | 0.165 ± 0.002 | 0.273 ± 0.022^***^ | 0.304 ± 0.004^***^ | 0.432 ± 0.013^***^ |
| **Thickness (mm)** | 0.020 ± 0.000 | 0.030 ± 0.000^NS^ | 0.040 ± 0.000^NS^ | 0.052 ± 0.005^*^ |
| **Diameter (cm)** | 8.463 ± 0.017 | 8.440 ± 0.018^NS^ | 8.358 ± 0.054^*^ | 8.487 ± 0.01^NS^ |
| **Density (g/cm3)** | 1.470 ± 0.009 | 1.632 ± 0.007 ^***^ | 1.384 ± 0.007^***^ | 1.460 ± 0.005^NS^ |

*Two-way ANOVA: multiple comparison*

*A vs. A+RE, A+Gly, A+Gly+RE*

*p<0.001 ***, p<0.01**, p<0.05*, p>0.05 ^NS^*

**Table S4. Moisture content, WST, and WVP**

|  | **Moisture (%)** | **WST (%)** | **WVP (%)** |  |
| --- | --- | --- | --- | --- |
| **A** | 15.91 ± 0.002 | 91.84 ± 0.006 | 5.369 ± 9.961 |  |
| **A+Gly** | 33.69 ± 0.003^***^ | 88.41 ± 0.006^***^ | 5.969 ± 9.013 ^NS^ |  |
| **A+RE** | 12.57 ± 0.001^***^ | 90.78 ± 0.114 ^NS^ | 5.345 ± 10.059 ^NS^ |  |
| **A+RE+Gly** | 28.36 ± 0.001^***^ | 86.54 ± 0.012 ^***^ | 7.033 ± 9.191^*^ |  |

*Two-way ANOVA: multiple comparison*

*A vs. A+RE, A+Gly, A+Gly+RE*

*p<0.001 ***, p<0.01**, p<0.05*, p>0.05 ^NS^*

**Table S5. Total Bacterial Count (TBC), log10 CFU/g**

|  | **Day 0** | **Day 7** | **Day 14** |
| --- | --- | --- | --- |
| **S (without biofilm)** | 3.421 ± 0.127 | 4.733 ± 0.083 | 5.425 ± 0.043 |
| **S+A** | 3.424 ± 0.112 ^NS^ | 4.366 ± 0.038 ^***^ | 4.243 ± 0.055 ^***^ |
| **S+A+Gly** | 3.439 ± 0.015 ^NS^ | 4.645 ± 0.041 ^*^ | 4.872 ± 0.086 ^***^ |
| **S+A+RE** | 3.467 ± 0.038 ^NS^ | 4.052 ± 0.026 ^***^ | 4.871 ± 0.135 ^***^ |
| **S+A+RE+Gly** | 3.484 ± 0.044 ^*^ | 4.510 ± 0.010 ^***^ | 5.026 ± 0.022 ^***^ |

*Two-way ANOVA: multiple comparison*

*S vs. S+A, S+A+Gly, S+A+RE, S+A+RE+Gly*

*p<0.001 ***, p<0.01**, p<0.05*, p>0.05 ^NS^*

**Table S6. Total Yeasts and Molds Count (TYMC), log10 CFU/g**

|  | **Day 0** | **Day 7** | **Day 14** |
| --- | --- | --- | --- |
| **S (without biofilm)** | 3.188 ± 0.023 | 3.734 ± 0.027 | 3.643 ± 0.019 |
| **S+A** | 3.192 ± 0.008 ^NS^ | 3.628 ± 0.047 ^***^ | 3.403 ± 0.031 ^***^ |
| **S+A+Gly** | 3.180 ± 0.007 ^NS^ | 3.695 ± 0.011 ^NS^ | 3.492 ± 0.017 ^**^ |
| **S+A+RE** | 3.232 ± 0.032 ^NS^ | 3.602 ± 0.013 ^***^ | 3.135 ± 0.144 ^***^ |
| **S+A+RE+Gly** | 3.283 ± 0.020 ^*^ | 3.697 ± 0.003 ^NS^ | 3.428 ± 0.053 ^***^ |

*Two-way ANOVA: multiple comparison*

*S vs. S+A, S+A+Gly, S+A+RE, S+A+RE+Gly*

*p<0.001 ***, p<0.01**, p<0.05*, p>0.05 ^NS^*
